# Superionic Conductivity in Ceria-Based Heterostructure Composites for Low-Temperature Solid Oxide Fuel Cells

**DOI:** 10.1007/s40820-020-00518-x

**Published:** 2020-08-29

**Authors:** Yifei Zhang, Jingjing Liu, Manish Singh, Enyi Hu, Zheng Jiang, Rizwan Raza, Faze Wang, Jun Wang, Fan Yang, Bin Zhu

**Affiliations:** 1 Jiangsu Provincial Key Laboratory of Solar Energy Science and Technology/Energy Storage Research Center, School of Energy and Environment, Southeast University, Nanjing, 210096 People’s Republic of China; 2 Department of Chemistry, Division for Pure and Applied Biochemistry, Lund University, Naturvetarvägen 14, 22362 Lund, Sweden; 3 Clean Energy Research Lab (CERL), Department of Physics, COMSATS University Islamabad, Lahore, Lahore, 54000 Pakistan

**Keywords:** Ceria-based heterostructure composite, Ceria–semiconductor, Energy band, Built-in field, Solid oxide fuel cell

## Abstract

Ceria-based heterostructure composite (CHC) has become a new stream to develop advanced low-temperature (300–600 °C) solid oxide fuel cells (LTSOFCs) with excellent power outputs at 1000 mW cm^−2^ level. The state-of-the-art ceria–carbonate or ceria–semiconductor heterostructure composites have made the CHC systems significantly contribute to both fundamental and applied science researches of LTSOFCs; however, a deep scientific understanding to achieve excellent fuel cell performance and high superionic conduction is still missing, which may hinder its wide application and commercialization. This review aims to establish a new fundamental strategy for superionic conduction of the CHC materials and relevant LTSOFCs. This involves energy band and built-in-field assisting superionic conduction, highlighting coupling effect among the ionic transfer, band structure and alignment impact. Furthermore, theories of ceria–carbonate, e.g., space charge and multi-ion conduction, as well as new scientific understanding are discussed and presented for functional CHC materials.

## Highlights

- Ceria-based heterostructure composite for novel semiconductor-ionic fuel cells.
- Superionic conduction at interfaces is associated with the crossover of band structure.
- Band alignment/bending resultant built-in field plays a significant role in superionic conduction.

## Graphical abstract

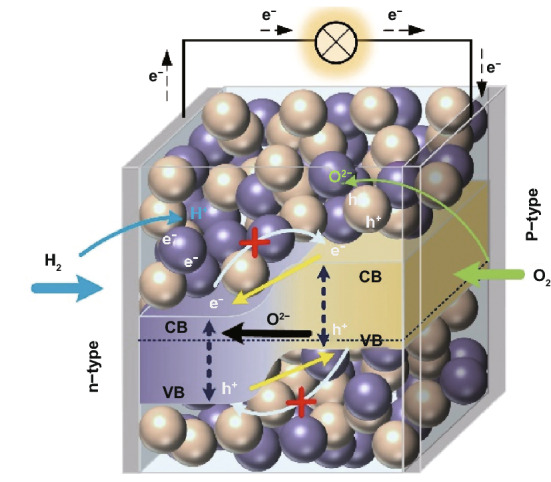

## Introduction

Solid oxide fuel cell (SOFC) has been proven to be one of the most efficient and promising energy conversion technologies because of its high efficiency and less or no environmental pollution [1]. However, conventional SOFCs need a high-temperature operation [2–4] for yttrium-stabilized zirconia (YSZ) electrolyte to reach the sufficient ionic conductivity, 0.1 S cm^−1^ at around 1000 °C [5]. As a crucial part of the SOFC, electrolyte undertakes the internal ionic conduction, which determines the performance of the SOFC system. In the last decades, commercial SOFC technology highly relies on the YSZ electrolyte for its stability, hence resulting in a huge cost due to the high temperature, which delays SOFC commercialization. Therefore, efforts have been devoted to lower down the operating temperatures of SOFCs. However, the conductivity of the conventional electrolytes in SOFC sharply collapses as temperature decreases from 1000 to 600 °C [6]. Hence, searching for novel electrolyte materials operating at lower temperature is a challenge to the current research paradigm [7].

Ceria-doped materials have gathered a considerable amount of interest as novel electrolytes materials for LTSOFC [8]. Various efforts have been made to understand and enhance the properties of ceria-based materials, like the structural doping by lower valent cations, e.g., ceria Ce^4+^ is doped with Sm^3+^ or Gd^3+^ earth elements to form a solid solution to maintain CeO_2_ fluorite structure*.* Remarkably, samarium-doped ceria (SDC) and gadolinium-doped ceria (GDC) have achieved a high ionic conductivity of 0.1 S cm^−1^ at 800 °C when compared to YSZ (*v.s.* 1000 °C for YSZ). However, the high operating temperature still remains for the usage of doped ceria. Another critical challenge related to doped ceria material is its instability in reducing conditions. For instance, under SOFC anodic condition, ceria-based electrolytes have significant electronic conduction, due to the reduction of Ce^4+^ to Ce^3+^, which leads to significant electrochemical leakage. Moreover, micro-cracking occurs in this reducing process of Ce^4+^ to Ce^3+^ due to a significant change in volume, which results in an operational failure of the cell. These problems are encountered as significant challenges for the doped ceria electrolytes [9].

An alternative to the ceria-based electrolytes is to introduce the second-phase materials, e.g., carbonates and semiconductors, to form a ceria-based heterostructure composites (CHCs), which are considered to be more stable in the reducing atmosphere. They have shown much better performance, e.g., ionic conduction of 0.1 S cm^−1^ reached below 600 °C and fuel cell power output at 1000 mW cm^−2^ level [10, 11]. Also, the anodic reduction of doped ceria electrolyte could be effectively avoided by CHCs. For example, the ceria–carbonate CHC system has a type of the core–shell structure, where ceria constitutes a core with the carbonate shell. Therefore, electrons localized in the ceria are not able to move from one ceria particle to another blocked by the carbonate shell; meanwhile, the carbonate shell can effectively prevent Ce^4+^ reduced to Ce^3+^, even if this situation occurs, the shell can also release the internal stress to avoid micro-cracking.

Benamira and Ringuedé et al. have demonstrated that ceria–carbonate systems showed stable operation from 1500 to over 6000 h [12, 13]. Many studies have proven that ceria–carbonate CHC materials show superior properties as electrolytes with the advantage of the multi- or hybrid-ion conduction, which enable excellent LTSOFC performances [9]. For example, excellent power output up to 1700 mW cm^−2^ was obtained at 650 °C [14], with a unique tri-ion of H^+^/O^2−^/CO_3_^2−^ conduction mechanism. In addition, 1200 mW cm^−2^ was achieved based on hybrid H^+^/O^2−^ ion conduction at a lower temperature (490 °C), attracting new research and development activities. Several reviews [4, 15–18] have tried to explain the superionic conduction in the ceria–carbonate CHC materials based on multi-ionic conduction at interface. However, the superionic conduction phenomena in ceria–carbonate CHC materials are not sufficient to fully understand its behavior. Moreover, the latest developments have demonstrated new interesting materials for ceria–semiconductor CHCs [10]. These new materials lead to the development of a single-component fuel cell (SCFC) and generation of new technology of semiconductor-ionic fuel cells (SIFCs).

**Fig. 1:**
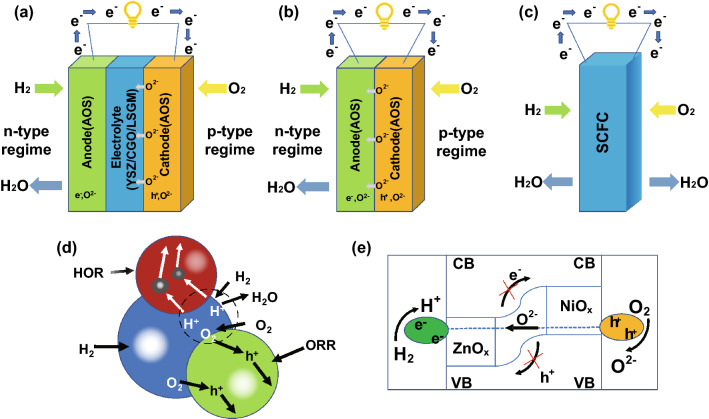
Schematic representation of different kinds of fuel cells: **a** conventional solid oxide fuel cells (SOFCs). **b** Double-layer fuel cell (DLFC), **c** SCFC, **d** SCFC–nanoredox fuel cell, **e** band alignment for SCFC

Zhu et al. invented a homogenous layer by mixing ionic conductor, e.g., SDC or GDC and semiconductor to integrate all functions from fuel cell anode, electrolyte and cathode into one component, so-called three-in-one technology, as highlighted by Nature Nanotechnology [19]. This single component or layer is a ceria–semiconductor CHC system. Compared to the conventional three-component fuel cell (Fig. 1a), SCFC, as shown in Fig. 1c, combines the electrodes and electrolyte into a homogeneous structure without using the electrolyte separator and three-component structure.

Removing the electrolyte layer could solve over a hundred-year challenge of the electrolyte limitation for SOFC commercialization. More importantly, this SCFC or “three-in-one” invention has sparked a new vision in the development of fuel cell science and technology. Singh et al. [20] pointed out that the fuel cell anode and cathode, using the amphoteric oxide, could be recognized as *n*-type and *p*-type regimes in H_2_ and air atmosphere, respectively, which was separated by an ionic conducting electrolyte separator, as shown in Fig. 1a. Practically, if we remove the middle electrolyte layer, it will turn to a *p*–*n* heterojunction device, as shown in Fig. 1b. This *p*–*n* heterojunction device can also realize the function of SOFCs without electrolyte layer as the separator, which therefore reduces the interfacial losses between electrodes and electrolyte, leading to a novel concept termed as double-layer fuel cell (DLFC) (Fig. 1b) [4, 21].

As a matter of fact, if we considered that the conventional anode/electrolyte/cathode device (Fig. 1a) could be compacted at the nanoscale, i.e., anode (*n*-type), electrolyte (intrinsic type) and cathode (*p*-type) components can be replaced by nanoscaled *n*, *i* (electrolyte as intrinsic semiconductor) and *p*-particles, as shown in Fig. 1d, this is then SCFC based on nanoredox working principle [21]. Nanoredox is a novel concept for a fuel cell. The overall nanoredox principle is presented in Fig. 1d [20], which is determined by the reaction of ions (H^+^ or O^2−^ or both). Because there is no electrolyte separator in the fuel cell device, this device is called electrolyte (layer)-free fuel cell (EFFC). In general, both H^+^ and O^2−^ may be transported to the corresponding cathode and anode side, the nanoredox reaction can take place on the surface of the nanocomposite particle, which consists of* n*,* p* and ionic conducting particles as shown in Fig. 1d. In case of both H^+^ and O^2−^, the reaction is

1 $$2{\mathrm{H}}^{ + } + {\mathrm{O}}^{{2 - }} = {\mathrm{H}}_{2} {\mathrm{O}}$$

If only H^+^ transported, it meets diffused atoms O or O_2_ from cathode side; the following reactions occur:

2 $$2{\mathrm{H}}^{ + } + {\mathrm{O}}({\mathrm{or}}\;1/2{\mathrm{O}}_{2} ) + 2{\mathrm{e}}^{ - } = {\mathrm{H}}_{2} {\mathrm{O}}$$

while in O^2−^ case the diffused H or H_2_ is involved,

3 $${\mathrm{H}}_{2} ({\mathrm{or}}\;2{\mathrm{H}}) + {\mathrm{O}}^{{2 - }} - 2{\mathrm{e}}^{ - } = {\mathrm{H}}_{2} {\mathrm{O}}$$

In all cases, the overall cell reaction process is:

4 $${\mathrm{H}}_{2} + {\mathrm{O}}~({\mathrm{or}}\,1/2{\mathrm{O}}_{2} ) = {\mathrm{H}}_{2} {\mathrm{O}}$$

This is the same as the common fuel cell process.

It is interesting to note that SCFC displays no short circuit or electrochemical leakage due to the band alignment and build-in-field (BIF) as presented in Fig. 1e [21]. This picture presents a new paradigm of research in SOFC and links from a macro-level device with anode, cathode and electrolyte components to micro-nanolevel, with each corresponding nanoparticle. It highlights a new understanding of SCFC and bridges the conventional fuel cell to novel SIFC developments.

The development of SCFC provides a new dimension of researches and developments on CHCs. Understanding the internal conduction of CHC from the micro-level is important. The state-of-the-art composition of the CHCs highlights new promising functionalities based on carbonate or semiconductor heterostructure composite materials with wide energy applications, which has not been brought into a review. Hence, this review aims to fill the existing gap in scientific knowledge via band theory and semiconductor aspect with updating new CHC materials. It further aims to provide a new methodology and strategy by introducing the band alignment and principle of the superionic conduction as a common scientific foundation of the CHC materials for advanced energy applications.

## Interfacial Superionic Conduction

**Fig. 2:**
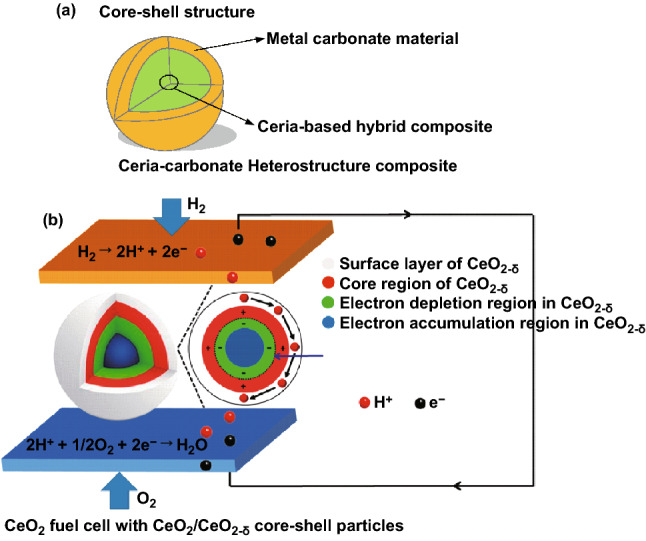
Illustration of core–shell structure of ceria-based heterostructure composite (CHC) systems: **a** ceria–carbonate CHC and **b** CeO_2_ core–shell particles as an electrolyte in CeO_2_/ CeO_2−δ_ core–shell structure. Reproduced with permission from Ref. [23]. Copyright 2019 American Chemical Society

Recent research in the field of LTSOFCs highlights the superionic conduction phenomenon in the CHC materials [4]. A typical CHC may be exampled using a core–shell structured ceria–carbonate composite, as shown in Fig. 2a. Between core and shell, the interface region plays a crucial role in ionic mobility and conductivity. Various ions are transferred through the interface form “high conductivity pathways” [22]. This core–shell structure is formed in ceria single phase, due to in situ formation of CeO_2_/CeO_2−δ_ core–shell particles in fuel cell environment, as shown in Fig. 2b. Proton shuttles are formed, leading to the interfacial/surface superionic conduction [23]. However, there are very few experimental analytical tools available to study the interface conduction directly [22]. Therefore, a wide variety of theoretical models and speculations have been proposed. Amongst all, the space charge model and lattice strain are two main mechanisms.

In the space charge region, one of the most important parameters is the space charge potential [24]. On the one hand, this parameter determines the distribution of electric field, which affects the transfer of ions. And on the other hand, the formation of the “high conductivity pathways” attributes to the lattice strain [25].

### Space Charge Model

The space charge region plays a vital role in understanding the theory of interfacial superionic conductivity. Wagner [26] and Maier [27–29] proposed the concept of space charge model. On applying this model to our CHC system, as a result of interfacial interaction, a space charge layer is generated at the interface, e.g., in SDC–carbonate system, the surface of SDC presents a positively charged region because of the enrichment of oxygen vacancies at the surface [30]. Since SDC is Sm^3+^ doped with Ce^4+^ to create oxygen vacancies, including surface doping, the oxygen vacancies accumulated at the surface of SDC will present the property of positively charged. In this case, the anions such as oxygen ions will be attracted to the positive charge region. Meanwhile, the cations will also be accumulated at the interface. Therefore, the conductivity of the composite will be enhanced due to the high concentration of the ions.

Based on Wagner and Maier’s models, the characteristic of the space charge region is described by the space charge potential (Δ*ϕ*). The Boltzmann statistics can be used to assist the calculation if the materials possess sufficiently dilute point defects. And the electrical potential can be calculated by the Poisson–Boltzmann differential [Eq. (5)] [31]:

5 $$\frac{{{\mathrm{d}}^{2} \phi }}{{{\mathrm{d}}x^{2} }} = - \frac{{ez_{j} c_{{j\infty }} }}{{\varepsilon _{0} \varepsilon _{{\mathrm{r}}} }}\exp \left( {\frac{{ - z_{j} e}}{{k_{{\mathrm{B}}} T}}\Delta \phi (x)} \right)$$

where *z*_*j*_*e* is the net charge, *c*_*j*∞_, the bulk concentration, *ε*_0_, the permittivity of free space, *ε*_r_, the relative dielectric constant, *k*_B_, the Boltzmann constant and *T*, the absolute temperature.

Among them, the charge carrier mobility can greatly influence the calculation [32]. Herein, we assume that all defective species have enough mobilities to redistribute in the space charge regions. Thus, Gouy–Chapman conditions can be introduced to this calculation if the order of the extension of the space charge region is the same as that of the Debye length, *L*_D_ [33], which may be expressed as Eq. (6):

6 $$L_{{\mathrm{D}}} = \sqrt {\frac{{\varepsilon _{0} \varepsilon _{{\mathrm{r}}} k_{{\mathrm{B}}} T}}{{2\left( {z_{j} e} \right)^{2} c_{{j\infty }} }}}$$

In a situation when these defects are not able to redistribute in the space charge layer, the decreasing defects (or effect) in the Poisson Boltzmann differential equation can be neglected and it can only be judged by the concentration of species *j*:

7 $$\frac{{{\mathrm{d}}^{2} \phi }}{{{\mathrm{d}}x^{2} }} = - \frac{{z_{j} ec_{{j\infty }} }}{{\varepsilon _{0} \varepsilon _{{\mathrm{r}}} }}$$

The Poisson Boltzmann differential equation can be substituted/simplified by Mott Schottky approximation if the space charge layer is much wider than Debye length [34].

8 $$\lambda ^{*} = \sqrt {\frac{{2\varepsilon _{0} \varepsilon _{{\mathrm{r}}} \Delta \phi (0)}}{{z_{j} ec_{{j\infty }} }}} = L_{{\mathrm{D}}} \sqrt {\frac{{4z_{j} e}}{{k_{{\mathrm{B}}} T}}\Delta \phi (0)}$$

where Δ*ϕ*(0) is regarded as the potential at the interface.

As can be seen from Eqs. (6) and (8), the width of the space charge layer is inversely proportional to the square root of the concentration of the point defects in bulk materials. Therefore, the space charge layer of heavily doped ionic conductors is extremely narrow, leading to an inapparent influence on the overall ionic conduction.

Furthermore, when the extent of the space charge layer is around the same as the Debye length, the Gouy Chapman analysis indicates that the change of the concentration of charged defects is significant where it is close to the interface. For the polycrystalline, the total conductivity will be affected by the space charge, when its grain size reaches the *L*_D_.

For the ceria–carbonate nanocomposite, the ions conductivity will be significantly affected by the space charge region due to nanoscale, so the above equations are meaningful for the CHC systems.

### Effect of Strain in CHC Systems

The effect of strain is another critical factor for the enhancement of ionic conduction in CHC systems. In particular, three strain effects are considered based on achieving excellent performance in CHC systems, including the facilitate of ionic transport, oxygen vacancy formation [35].

**Fig. 3:**
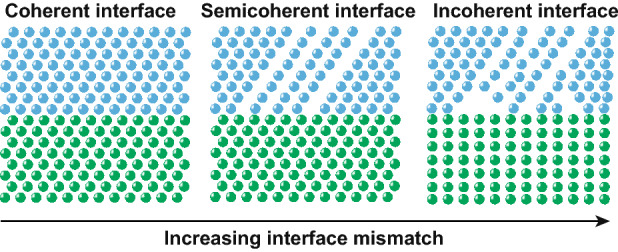
Order of lattice mismatch with different interfacial structures: coherent interface, semicoherent interface and incoherent interface. Reproduced with permission from Ref. [36].Copyright 2008 Royal Society of Chemistry

Usually, strain often forms in a heterostructure due to the lattice mismatch of two adjacent phases, which relates to temperature, the lattice mismatch and the thickness of thin-film [35]. According to the degree of the mismatch, the interface can be classified into three types, as shown in Fig. 3, called coherent, semicoherent and incoherent [25, 36, 37].

With increasing interface mismatch, there will be a misfit dislocation of the network of two adjacent phases to decrease the strain at the interface. Thus, a coherent interface is beneficial to fabricate and control the lattice strain due to the compensation of the strain.

**Fig. 4:**
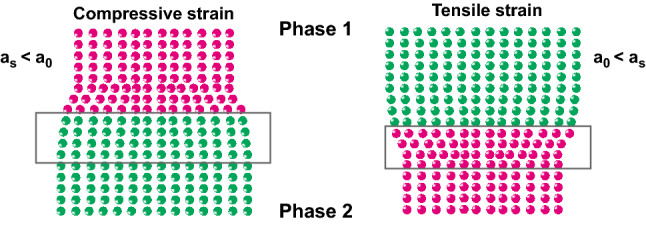
Compressive strain and tensile strain at the interface

Typically, different types of lattice strain at the interfaces could be described as the compressive strain and the tensile strain, respectively, as shown in Fig. 4, which are determined by Eq. (9) [38–40]:

9 $$\varepsilon = \frac{{a_{{\mathrm{s}}} - a_{0} }}{{a_{0} }}$$

where *a*_s_ is the strained lattice parameters; *a*_0_ is the unstrained lattice parameters. And the tensile strain will be formed if *a*_s_ > *a*_0_, while the compressive strain will be formed at the interface if *a*_s_ < *a*_0_.

And Schichtel et al. proposed a quantitative Eq. (10), which could be used to express the relationship between strain and ionic conductivity [41].

10 $$\ln \left( {\frac{{\sigma _{t} }}{{\sigma _{0} }}} \right) \cong \frac{2}{3}\frac{{\Delta V_{{V_{O}^{{ \bullet \bullet }} }}^{M} }}{{RT}}\frac{Y}{{1 - v}}\varepsilon$$

where *T* is the absolute temperature;* Y*, the Young’s modulus; $v$, the Poisson ratio;* ε*, the interfacial strain resulting from lattice mismatch; and $\Delta V_{{V_{O}^{{ \bullet \bullet }} }}^{M}$ the migration volume of $V_{O}^{{ \bullet \bullet }}$.

The lattice strain is considered as a factor affecting the ionic migration, especially the tensile strain. Based on DFT model, Souza et al. investigated the effect of the lattice strain and calculated the migration energy for oxygen vacancy [42]. The results reflected that the ionic conductivity could increase 4 orders of magnitude with a 4% tensile strain at 500 K. Conversely, according to the results of molecular dynamics (MD) simulation, Rushton et al. found that the oxygen diffusion coefficient only enhanced 15 times at 1900 K [43], which seems not apparent. Furthermore, the migration energy of all dopants was studied. It is interesting to find that a tensile strain of 5% will lead to a reduction of the migration energy from 0.96 to 0.182 eV. Therefore, the tensile strain is indicated to be a significant factor in promoting ionic transport.

Besides, the lattice strain could promote the formation of an oxygen vacancy, especially of the CeO_2_ materials [25, 44]. Commonly, the formation energy of oxygen vacancy (*E*_f_) could be expressed by Eq. (11) [45, 46]:

11 $$E_{{\mathrm{f}}} = E_{{\mathrm{V}}} - E_{{{\mathrm{surf}}}} + \frac{1}{2}E_{{{\mathrm{O}}_{2} }}$$

where *E*_v_ is the system energy of one oxygen vacancy, *E*_surf_, the total energy of the bare surface, and $E_{{{\mathrm{O}}_{2} }}$, the total energy of an O_2_ molecule.

Using DFT model, Ahn et al. demonstrated that the formation energy of oxygen vacancy is strongly related to lattice strain [44]. And compared with compressive strain, tensile strain is more beneficial for increasing oxygen vacancy. In addition, Aidhy et al. claimed that tensile strain could decrease the formation energy of oxygen vacancy and affect the stability for CeO_2_/ZrO_2_ and other oxides [47], which led to the tendency of the generation of oxygen vacancy, especially in CeO_2_.

### Multi-ionic Conduction

Hybrid H^+^/O^2−^ or multi-ionic conduction (M^+^/H^+^/O^2−^/O) has been reported for ceria–carbonate CHC systems [48]. Hybrid H^+^/O^2−^ conduction behavior is common nature for the ceria–carbonate CHCs with good LTSOFC performances [49]. Zhu et al. studied the performance of SDC–LiKCO_3_ [50] and found that the conductivity of this composite is one magnitude higher than that of pure SDC. Furthermore, the work suggested that the enhanced conductivity attributes due to three kinds of charge carrier O^2−^, CO_3_^2−^, H^+^ in SDC–LiKCO_3_. Also, Huang et al. have reported that the possible multi-ionic conduction resulted in excellent fuel cell performances [51]. Many efforts have been devoted to understanding the mechanism of the multi-ionic conduction, e.g., Wang et al. proposed the “Swing Model” and tried to explain the transport of protons [48]. Recently, the conduction mechanism in oxide–carbonate-based electrolytes has been reviewed by Ricca et al., which is based on the first-principles modeling [22] and density functional theory (DFT).

The concept of DFT originated from the Thomas–Fermi model [52] and further the Hohenberg–Kohn principle laid a solid foundation for DFT application with its first and second principles [53]. In most cases, DFT gives very satisfactory results compared with other theoretical methods to solve the multi-body problem in quantum mechanics, and the solid-state calculation is less expensive than experiments [54].

Based on the accurate and reliable description of the structural and electronic properties of both SDC and LiKCO_3_ phases reported, the periodic interfacial model was established. In this process, different elements and positions can be substituted according to the doping concentration and their natural properties [22]. Then, the math models are constructed by Born–Oppenheimer approximation, Hartree–Fock equation and electron density functional theory [55]. The detailed information of the calculation can be found in Appendix. About the geometry optimization process, only atoms in the nearest position of the defects were allowed to relax, until the maximum, root mean square atomic forces and displacements were simultaneously less than selected value, respectively [56].

Here, we summarize the recent explanation of the transport mechanisms from the perspective of DFT.

#### Cations of Carbonate

Although the enhancement of fuel cell performance does not depend on the migration of cations, still the role of cations is beneficial. According to Maier’s theory of space charge regions, the cation of carbonate can stabilize itself by adsorption on the oxide, which will further increase the cation vacancy of the carbonate phase near the boundary [57, 58]. The reaction is as follows:

12 $${\mathrm{M}}_{M}^{x} + V_{s} \to {\mathrm{M}}_{s}^{\cdot} + V_{M}^{\prime}$$

**Fig. 5:**
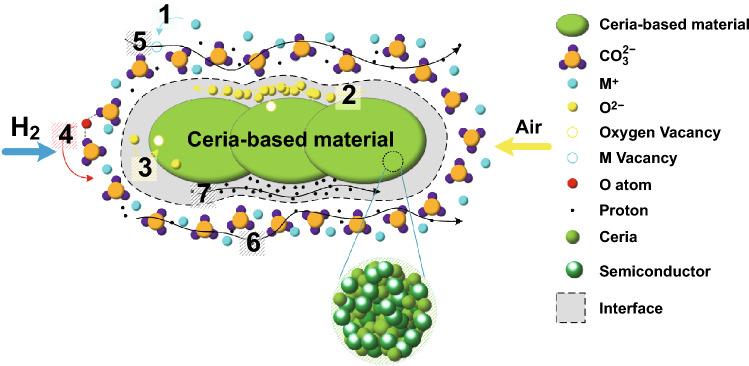
Cogwheel mechanism for the migration of (1) cations (M^+^) of the carbonate, (2) oxide (O^2−^) anions of the oxide at the interface, (3) the oxide anions at bulk, (4) neutral O in the carbonate (CO_3_^2−^), (5) proton with the help of M^+^ vacancy, (6) proton in carbonate and (7) the bond chain at the interface in ceria-based material

During the reaction, the cation M^+^(Na^+^/K^+^/Li^+^) hops from its original lattice site to a nearby vacancy, leaving a neutral M_s_^•^ and a vacancy of M^+^ (see position 1 in Fig. 5). Ricca et al. claimed that the function of $V_{M}^{\prime}$ was attributed to neutral M Frenkel pairs (M_FP_) [22]. The formation energy of Li_FP_ in the composite phase (1.07 –1.26 eV) is lower than that in the pure carbonate phase (1.79 eV) [59]. This result indicates that the formation of the interface can indeed change the defective chemical properties of carbonate facies as the interface area between the core and shell has a stable interaction. Maier's model assumes that M_s_^•^ is stable, which separates at the interface and the enhanced conductivity is due to vacancy diffusion in the space charge layer. Therefore, only the most stable M_FP_ structure is considered to study the diffusion of cation vacancy. In the case of Li vacancy, Li diffusion corresponds to repeated process of the exchange of positions of a lattice Li^+^ and vacancy. Then, Li_i_^•^ is fixed at the interface, and relaxed scan calculations are performed for a vacancy that shows Li^+^ diffuses into the Li_FP_ from its initial position along the selected path according to a direct hopping mechanism. Ricca et al. show that the vacancy diffusion barrier (0.23 eV) in the composites is much smaller than the vacancy diffusion barrier (> 10 eV) in the pure LiKCO_3_ [22]. Therefore, the formation of a composite material interface determines the decrease of the defect formation energy (accompanied by M_FP_) and the decrease of the vacancy diffusion barrier. Besides, the interaction between carbonate phase and ceria core leads to the change of M^+^ transport mechanism, because the vacancy jumping is forbidden in pure carbonate material, while the M^+^ vacancy can easily spread in the space charge field. Furthermore, the diffusion of M^+^ can make a contribution to proton transport as it can play the role of an intermediate medium for proton transport, which results in superionic transition.

#### ***O***^***2−***^*** Ion Conduction***

O^2−^ ion conduction is a traditional conduction mechanism and it occurs in the structure via oxygen vacancy. However, the mechanisms in the ceria–carbonate CHC are different from the O^2−^ ion bulk conduction. For example, Huang et al. proposed that O^2−^ ions can accumulate on the surface of oxide particles, which result in higher oxygen vacancy concentration due to interfacial interaction as described by Eq. (13) [59]:

13 $${\mathrm{O}}_{{\mathrm{O}}}^{{\mathrm{X}}} + {\mathrm{V}}_{{\mathrm{s}}} \to {\mathrm{O}}_{{\mathrm{s}}}^{\prime\prime} + {\mathrm{V}}_{{\mathrm{O}}}^{{..}}$$

where the O^2−^ ion conduction mechanism can be divided into two parts: bulk conduction (see position 3 in Fig. 5) and interface conduction (see position 2 in Fig. 5). In order to simulate the mechanism of a higher concentration of defects/ions near the phase boundary from DFT, the usual method is to introduce an O^2−^ ion and two interstitial M_i_^•^ ([2M_i_^•^ − O^2−^]) at the interface to simulate simultaneous adsorption of negatively charged and positively charged species.

Raza et al. have demonstrated that the conduction via oxygen vacancy in the bulk oxide is much slower than at the interface between carbonate and ceria CHCs [60] due to higher hopping barrier in bulk than in the interface. Another explanation for the conductivity enhancement is based on Maier’s space charge layer theory [57, 58]. A space charge layer can be formed at the phase boundaries due to interfacial interaction, which results in accumulation of the oxygen ions at the surface of the oxide and a high M^+^ ion concentration at the carbonate surface. The higher concentration of the defects/ions near the phase boundaries compared with the bulk may essentially constitute superionic pathways at the boundary/interface between two phases.

In addition, the O atom migration at the ceria-based carbonate interface is worth considering as carbonate which may enhance the oxygen reduction process in SOFC by a unique mechanism of O atom transport in the carbonate phase. The transport mechanism is a kind of Cogwheel mechanism, which includes continuous breaking and regeneration of the O–CO_3_^2−^ bond (see position 4 in Fig. 5):

14 $${\mathrm{CO}}_{{\mathrm{4}}}^{{{\mathrm{2}} - }} + {\mathrm{CO}}_{{\mathrm{3}}}^{{{\mathrm{2}} - }} \to {\mathrm{CO}}_{{\mathrm{3}}}^{{{\mathrm{2}} - }} - {\mathrm{O}} - {\mathrm{CO}}_{{\mathrm{3}}}^{{{\mathrm{2}} - }} \to {\mathrm{CO}}_{{\mathrm{3}}}^{{{\mathrm{2}} - }} + {\mathrm{CO}}_{{\mathrm{4}}}^{{{\mathrm{2}} - }}$$

#### Peroxide (O_2_^2−^) ***Ion Conduction***

**Fig. 6:**
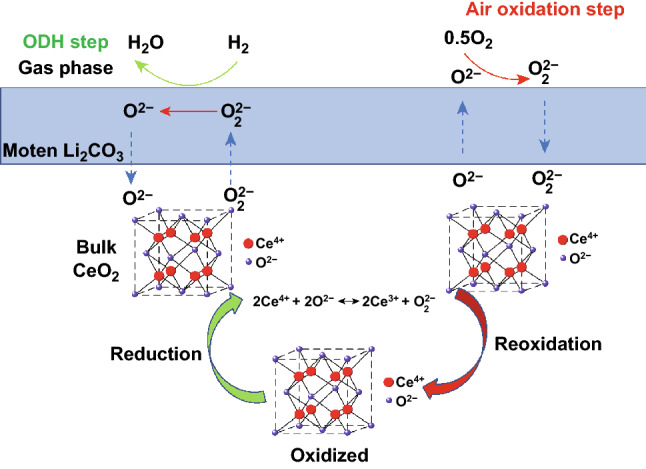
Illustration of the mechanism of the peroxide (O_2_^2−^) ion conduction for ceria–carbonate heterostructure composite

The conduction of O_2_^2−^ ion is a novel mechanism, which is attributed to a circular reaction, as shown in Fig. 6. Recently, Gao et al. demonstrated that the O_2_^2−^ ion conduction plays a crucial role in the LSF(La_0.8_Sr_0.2_FeO_3_)–Li_2_CO_3_ composite and provides a pathway for O_2_^2−^ ions through the DFT [61]. In this regard, we proposed a similar conduction mechanism for ceria–carbonate CHC system. In this case, there will be three vital reactions promoting the transport between ceria and carbonate, as shown in Fig. 6.

First, O_2_^2−^ ion forms in the lattice of the CeO_2_ due to reduction of Ce^4+^, as shown in Eq. (15):

15 $$2{\mathrm{Ce}}^{{4 + }} + 2{\mathrm{O}}^{{2 - }} \leftrightarrow 2{\mathrm{Ce}}^{{3 + }} + {\mathrm{O}}_{2}^{{2 - }}$$

Then, this O_2_^2−^ ion will cross the molten carbonate and transport to the surface of the molten carbonate. Furthermore, oxidative dehydrogenation (ODH) will take place as shown by Eq. (16):

16 $${\mathrm{H}}_{2} + {\mathrm{O}}_{2}^{{2 - }} \to {\mathrm{H}}_{2} {\mathrm{O}} + {\mathrm{O}}^{{2 - }}$$

In addition, at the surface of the carbonate, a re-oxidation step will take place. The gaseous O_2_ will react with the O^2−^ to form O_2_^2−^ ion as shown in Eq. (17):

17 $${\mathrm{O}}_{2} + 2{\mathrm{O}}^{{2 - }} \to 2{\mathrm{O}}_{2}^{{2 - }}$$

Then, the O_2_^2−^ forming at the surface of the carbonate will migrate back to the ceria core. Here, the Ce^3+^ will be re-oxidized by the O_2_^2−^ and replenish the active lattice oxygen species as shown in Eq. (18):

18 $$2{\mathrm{Ce}}^{{3 + }} + {\mathrm{O}}_{2} ^{{2 - }} \leftrightarrow 2{\mathrm{Ce}}^{{4 + }} + 2{\mathrm{O}}^{{2 - }}$$

#### Extrinsic Species: Proton (H^+^) Conduction

H^+^ transportation plays an important role in the ionic conduction in ceria–carbonate CHCs and contributes more significantly than O^2−^ ion in CHCs. Zhu et al. proposed a H^+^ conduction mechanism for the first time [62], and further, they demonstrated that temporal bonding between H^+^ and CO_3_^2−^ ion plays a vital role in this process (see position 6 in Fig. 5). Overall, three kinds of motions of proton in carbonate should be considered [63]: (1) H^+^ rotates around the O^2−^ ion forming some kind of interaction bond with the O^2−^ ion; (2) H^+^ migrates from one O^2−^ ion to another, while both are located at the same carbonate ions; (3) H^+^ migrates from one O^2−^ to another of the different carbonate ions. From the result of Lei et al. [63], it is clear that the small energy barrier (0.18 eV) plays a role in H^+^ transfer; nevertheless, the rotation needs a higher energy barrier (about 0.35 eV), which means the latter is the rate-determining step. However, Ricca et al. demonstrated that H^+^ is more energetic at the interface [22]. Thus, the investigation at the interface is crucial. Based on the work of Huang et al. [51], a simple linear direct hopping mechanism has been proposed. In this assumption, H^+^ is regarded as an interstitial species at the interface, which diffuses from the initial position to the final with the most stable configuration (see position 5 in Fig. 5) as explained in Eq. (19):

19 $${\mathrm{H}}_{{\mathrm{f}}}^{{\mathrm{X}}} + {\mathrm{V}}_{{\mathrm{M}}}^{\prime} \to {\mathrm{H}}_{{\mathrm{M}}}^{{\mathrm{X}}} + {\mathrm{V}}_{{\mathrm{i}}}^{\prime}$$

In addition, the H^+^ transport can be affected by the interaction between carbonates and the oxygen species on the surface.

**Fig. 7:**
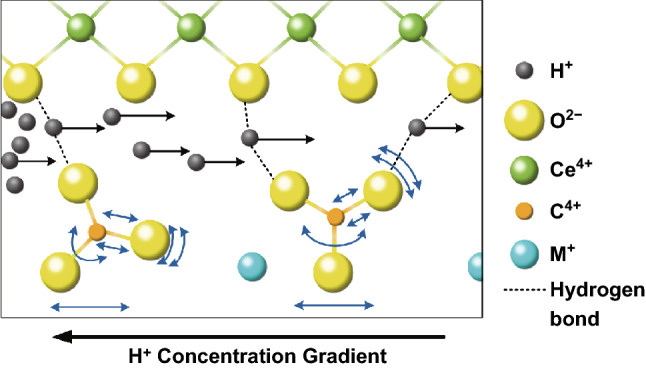
Proposed “Swing Model” in samarium-doped ceria (SDC)–Na_2_CO_3_ nanocomposite. Reproduced with permission from Ref. [48]. Copyright 2011 Elsevier

In order to explain the transport of H^+^, Zhu and Mat proposed that proton could move from the initial position of the O surface site to another site through the conduction of H-bond chain at the interface [64] (see position 7 in Fig. 5). Further, Wang et al. introduced an empirical “Swing Model” [48] to explain the H^+^ conduction mechanism in ceria–carbonate CHCs. In this process, carbonate serves as a bridge through a continuous hydrogen bond breaking and formation for proton to migrate from one hydrogen bond to another, as shown in Fig. 7.

## Effect of Energy Band

Conventional interfacial conduction is attributed to the formation of a space charge region due to the mismatch between two different lattices. However, it is not enough to explain the mechanism of the enhanced ionic conductivity for CHCs. Here, we introduce energy band and build-in-field to explain this interesting interfacial superionic conduction.

Energy band (EB) effect firstly appeared in CHC based on ceria–semiconductor systems [10, 65]. As a matter of fact, EB is a universal trend to describe not only semiconductors material but also other materials, like metal and insulator. All these materials can be differentiated by band structures and specifically by bandgap values. With various characterizations of semiconductor materials, conduction band (CB) and valence band (VB) play an irreplaceable role in determining the conductivity of heterostructure composite. Similarly, as a function of CB and VB, EB structure also affects the electron–hole separation and relevant electron conductivity. Several reports [66, 67] have claimed that the modified band structure is helpful in improving fuel cell performance. In this section, the modification mechanism of the EB structure in CHC is analyzed, including band alignment and built-in field. Moreover, mathematic descriptions of ionic conductivity enhancement with reducing activation energy, enhancing ion concentration and built-in-electric-field effects are proposed.

### Mechanism of Band Alignment

**Fig. 8:**
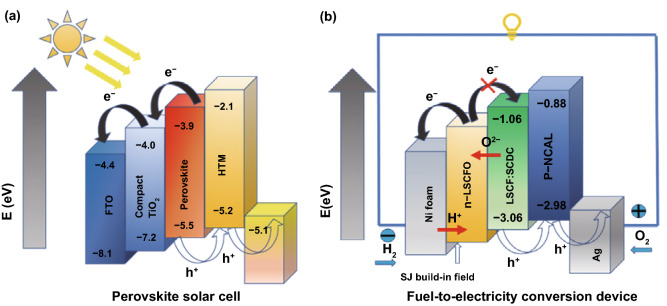
Energy level diagram of **a** perovskite solar cell, **b** fuel-to-electricity conversion device inspired by the perovskite solar cell structure. Reproduced with permission from Ref. [11]. Copyright 2016 Elsevier

The band structure and alignment principles have been explored for fuel cell applications by Zhu et al. [68]. Commonly a doped ceria (SDC or GDC) forms a heterostructure when semiconductor phase is introduced, which is employed to replace the electrolyte in fuel cell devices for SIFCs. Since such bulk heterostructure composites with a semiconductor phase can easily reach superionic conduction with ionic conductivity > 0.1 S cm^−1^, so more and more semiconductor composites have been applied to function as an electrolyte for advanced LTSOFCs application. Surprisingly, the semiconducting phase introduced to CHC system did not cause any electronic conduction, and the challenge remains in understanding the science behind. In this regard, the band alignment, as shown in Fig. 8, is proposed to play a crucial role in this internal electronic process [11].

**Fig. 9:**
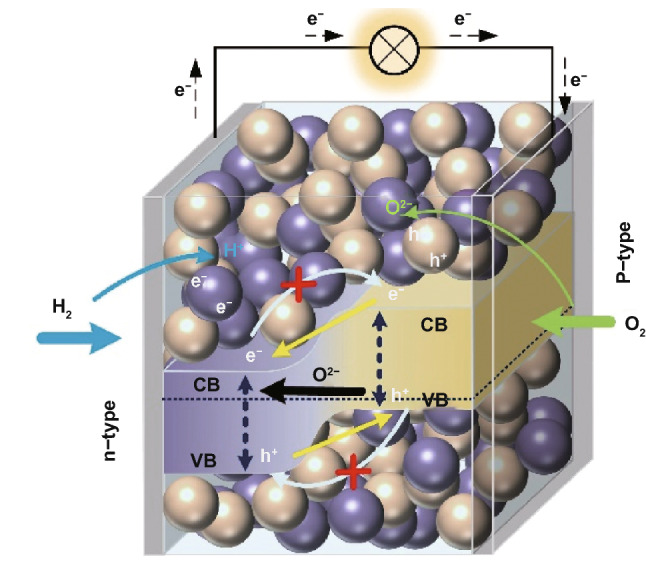
Band structure and alignment for a bulk *p*–*n* heterojunction SIFC device

Proper band design is very useful for enhancing the performance of CHCs. As shown in Fig. 8, band bending phenomena occur when one semiconductor comes in contact with another. Thus, formed band bending results from the close contact between two semiconductors having different CB and VB [21]. In thermal equilibrium, electrons will flow from the high Fermi level to the low one due to the different Fermi energy levels of these two semiconductors. Meanwhile, the build-in-field (BIF) is generated to form bulk *p*–*n* heterojunction, e.g., in the ZnO_*x*_ and NiO_*x*_ system the BIF directs from ZnO_*x*_ to NiO_*x*_. Then, the high Fermi level will move down with the CB and VB, while the low Fermi level with CB and VB will move up until these two Fermi levels are aligned with each other. Next, the formation of a potential barrier and BIF will resist the movement of the electrons and hole. The band bending and BIF can modulate the electron/hole migration across the heterojunction for inhibiting electron transport through the electrolyte by providing an additional electric field. Here, *p*–*n* heterojunction is formed with the contact between a *p*-type semiconductor and *n*-type semiconductor. And the CB and VB of *p*-type are both higher than those of *n*-type, respectively. Thus, electrons in *n*-type semiconductors tend to migrate to *p*-type semiconductors, leaving a positively charged region nearby [69]. Likewise, holes in *p*-type semiconductor tend to move to *n*-type and leave a negatively charged region until reaching the Fermi level equilibrium of the system. The surface of the *p*–*n* heterojunction is the area of charge, i.e., BIF. When the cell starts to work (when the energy is equal to or higher than the bandgap width), electron–hole pairs are produced. Electrons in a *p*-type semiconductor migrate to CB in *n*-type semiconductor, and holes in *n*-type semiconductor to VB in *p*-type semiconductor (Fig. 9). Because of the synergy of the BIF, the probability that the electrons migrate from *n*-type region to *p*-type region is prevented, thus inhibiting the possibility of short circuit. Also, BIF will improve on the ionic conductivity via positive Coulomb force. Furthermore, Zhang et al. demonstrated that in the case of an applied electrical field, the potential in the space charge region decreases from *n*-type region to *p*-type region and the potential energy of the electron transition increases from *n*-type region to *p*-type region. The *p*-type region moves up relative to the *n*-type region; band bending is formed at the junction surface; thus, extra barrier for electron transition from *n*-type to *p*-type could also take place [69].

Consequently, the semiconductor heterojunction has a significant influence on the overall CHC property and fuel cell performance. So, semiconductor characteristics between the hybrid phases are responsible for the change in an electron transport mechanism. Further, it can also improve the ionic conduction of CHC through a BIF mechanism described in the next section.

### Built-in Field (BIF)

One of the most distinct characteristics and advantages of introducing the band theory into CHCs is the BIF, which can promote ionic transport with significantly enhanced conductivity. According to Arrhenius relation:

20 $$\sigma = \sigma _{0} \exp \left( { - \frac{{E_{{\mathrm{a}}} }}{{k_{{\mathrm{B}}} T}}} \right)$$

where *E*_a_ is the activation energy of conduction; *k*_B_ is the Boltzmann constant; *σ*_0_ is the pre-exponential factor, which is related to the concentration of charge carriers. There are three aspects for BIF to promote ionic conductivity: (1) decrease the activation energy to promote the migration of the ions; (2) increase the mobile ion concentration; and (3) drive directly and assist ions’ transport. These are further analyzed in the following.

#### Ionic Conductivity and Activation Energy

The concept of activation energy was first proposed by Arrhenius, which was applied to describe the minimum energy required for a reaction. Here, the activation energy is used to refer to the minimum energy needed for the migration of ions. The activation energy of the ionic conduction is the sum of the structural association energy (*E*_A_) and the migration energy (*E*_m_):

21 $${E_{{\mathrm{a}}} = E_{{\mathrm{A}}} + E_{{\mathrm{m}}} }$$

The *E*_A_ reflects the bond of the ions and vacancies; thus, *E*_A_ can be reduced due to the thermal activation at high temperatures [11, 70]. BIF in the interface region/space charge region (a few nanometer scale) is in order of 10^6^ V m^−1^ [71, 72]. Such a strong electric field can certainly activate ions from static to mobile, i.e., break the bond so that *E*_A_ is not necessary. Therefore, the total activation energy of ionic transportation may be reduced to sole *E*_m_, i.e., the ions’ migration energy. On this basis, a Columbic interaction model to describe the CHC interfacial superionic conduction is proposed as [73]:

22 $$E_{{\mathrm{m}}} = k\frac{1}{{4\pi \varepsilon _{0} \varepsilon _{{\mathrm{r}}} }}\frac{{Qq}}{r}$$

where *E*_m_ is the migration energy; *Q* or *q* is the quantity of electric charge; *ε*_r_ is the relative dielectric permittivity; *ε*_0_ is the dielectric permittivity of vacuum; *k* is the Columbic constant, 9.0 × 10^9^ Nm^2^/C^2^; *r* is the distance between two opposite charges.

In SDC–Na_2_CO_3_, the theoretical value of the oxygen ion migration energy can be calculated as 0.2 eV at the interface, which is much smaller than that of bulk conduction in single-phase ceria (1.0 eV) [74]. While the migration energy of proton is also calculated to be 0.1 eV at the interface of SDC–Na_2_CO_3_, compared with the bulk single-phase material, the migration energy decreases significantly, which promotes the conduction of the ions at the interface leading to superionic conduction.

#### Mobile Ions Concentration

Higher mobile ion concentration will lead to higher ions’ conductivity according to Arrhenius relation, as the pre-exponential factor is proportional to the concentration of mobile ions. In CHCs, the BIF strong field interaction can activate ions from static to mobile, e.g., the cations are partially drawn out of the crystal and pushed into interstitial sites [75], both surface vacancy and interstitial ions are created at the same time. Therefore, the concentration of charge carriers (vacancies, interstitials) will be significantly enlarged in the space charge region of CHCs. In this concern, metal carbonates (M_2_CO_3_, M = Li, Na, K) or metal semiconductor (MOS), e.g., NiO_*x*_, CoO_*x*_, CuO_*x*_, FeO_*x*_ or their lithiated compounds, e.g., LiNiO_2_, LiCoNiO_2_ and LiCoAlO_2_, are the excellent second phase to form the heterostructure with ceria to produce extremely highly mobile ion concentrations in space charge region of the CHC systems.

#### BIF Assisting Ion Transport

**Fig. 10:**
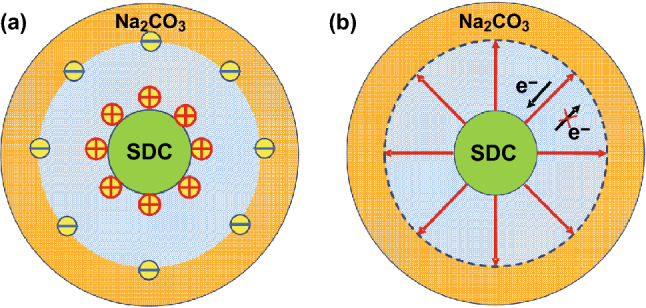
Built-in-field (BIF) effect between SDC (samarium-doped ceria) and Na_2_CO_3_

As shown in Fig. 10, SDC surface is positively charged as discussed above (Fig. 10a); thus, the direction of the resulted BIF will point toward the amorphous sodium carbonate phase from the SDC crystalline core (Fig. 10b), which blocks the migration of electrons. Thus, the electron located on the surface of the SDC cannot migrate to the surface of Na_2_CO_3_ and avoids the electronic internal short circuit.

Here, we consider the intrinsic O^2−^ and H^+^ at the interface, the electrochemical potential of X (O^2−^/H^+^) species, *ϕ*_*x*_, consists of the chemical potential (*ϕ*_*c*,*x*)_ and electrical potential (*ϕ*_*e*,*x*)_ as follows:

23 $$\phi _{x} = \phi _{{c,x}} + N_{x} F\phi _{{e,x}}$$

where *N*_*x*_ is the effective charge of O^2−^/proton and *F* is Faraday’s constant, 96,500 C/mol. Taking the virtual force on the particle to be the negative gradient of *ϕ*_*x*_ rather than of *ϕ*_*e*,*x*_ alone:

24 $$F_{i} = - 1/N_{{\mathrm{a}}} ({\mathrm{d}}\phi _{x} /{\mathrm{d}}x)$$

It is obvious that the flux *x* is a function of the electric field d*ϕ*_e,x_/d*x*:

25 $$f_{X} = - C_{x} B_{x} /N_{{\mathrm{a}}} ({\mathrm{d}}\phi _{x} /{\mathrm{d}}x) = - C_{x} B_{x} /N_{{\mathrm{a}}} ({\mathrm{d}}\phi _{{c,x}} /{\mathrm{d}}x + {\mathrm{d}}\phi _{{e,x}} /{\mathrm{d}}x)$$

where *B*_*x*_ is absolute mobility and *C*_*x*_ is the concentration of *x* species.

As can be seen from Eq. (25), the electric force will offset the effects of a large concentration gradient in the opposite direction, which helps to push O^2−^/H^+^ toward Na_2_CO_3_/SDC. Therefore, in the fuel cell condition, the extrinsic diffused H^+^ ion from the Na_2_CO_3_ will probably be located at the interface space region due to the positive “ + ” surface of the SDC. For this reason, proton will be forced to move along the surface or at the interface between the SDC and Na_2_CO_3_. Meanwhile, the movement of electrons will be suppressed under the influence of BIF. Further, this BIF electric field as an extra driving force is added to the chemical potential from the ion concentration gradient, which assists the migration of the ions.

26 $$F = q\left( {\frac{{{\mathrm{d}}\phi _{{c,x}} }}{{{\mathrm{d}}x}} - \frac{{{\mathrm{d}}\phi _{{e,x}} }}{{{\mathrm{d}}x}}} \right)$$

Here, the electrical force generated by BIF will offset part of the effect of the chemical potential.

In conclusion, it is evident that BIF plays a crucial role in the conduction of CHC systems from three aspects. Thus, constructing a proper BIF through space charge or band alignment is important to improve the properties of CHC fuel cells. Further, this theory will be applied to explain the conduction mechanism of ceria–carbonate CHC systems.

### Mechanisms of Ceria–Carbonate CHC Systems

Based on the above description, the concept of phase junction will be further introduced to explain the conduction mechanisms of ceria–carbonate CHC systems. It is known that different crystal phases may possess different energy band structures; therefore, it is possible to form a homojunction between different phases in one semiconductor, which is called phase junction or surface junction [69].

#### Phase Junction

**Fig. 11:**
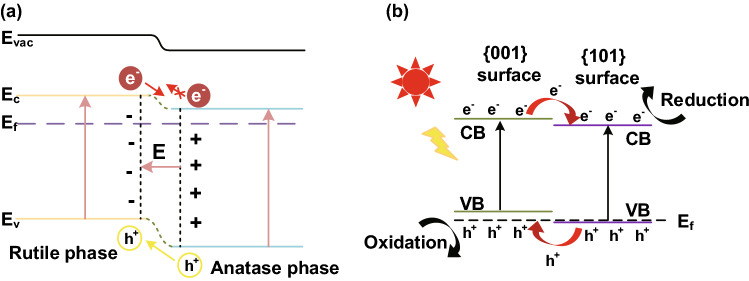
**a** Illustrations of the proposed band alignment between rutile and anatase. Reproduced with permission from Ref. [78]. Copyright 2015 Elsevier;** b** {001} and {101} surface heterojunction. Reproduced with permission from Ref. [77]. Copyright 2014 American Chemical Society

A typical paradigm of phase junction is formed by TiO_2_, as revealed by Yan et al. [76]. As shown in Fig. 11a, the overall band structure of anatase phase is slightly lower than that of rutile phase. Therefore, it allows holes to accumulate in rutile phase and electrons to accumulate in anatase, which is similar to the mechanism of *p*–*n* heterojunction, especially in the band alignment directions of holes and electrons. This is called as the *pseudo p–n junction* which can also generate a BIF with the same effects by accelerating ionic conduction and suppressing electrons transport. In addition, Yu et al. [77] proposed that the phase junction may generate even in a single-phase particle, e.g., there are differences in the energy band structure of anatase TiO_2_ between the {101} facets and {001} facets, as shown in Fig. 11b.

Compared with traditional *p*–*n* heterojunction, the novel phase junction can realize hole–electron pairs separation without two or more semiconductors. Therefore, it is reasonable to conclude that the most significant effect in the formation of heterojunction is not different kinds of semiconductors, but the implementation of band alignment. Likewise, the working principles of ceria–carbonate CHC system can also be explained by phase junction.

#### Carbonate Modified Band Structures

**Fig. 12:**
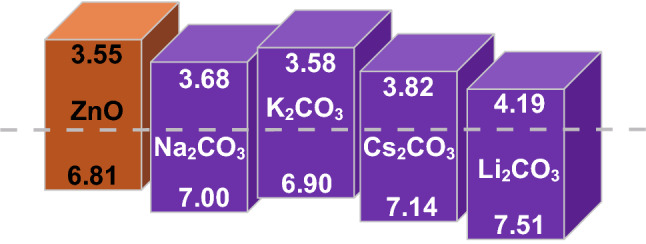
Energy levels of ZnO ripple layers incorporated with various metal carbonate materials. Reproduced with permission from Ref. [66]. Copyright 2016 Royal Society of Chemistry

CB and VB can be remarkably modified after incorporation with carbonate, as shown in Fig. 12. It can be seen from Fig. 12 that the band structure of ZnO exhibits an overall decrease, after incorporating carbonate into pure ZnO [66]. Jeong et al. [67] pointed out that compared with pure ZnO, carbonate could lead to a visible decline of the band structure. Therefore, it is reasonable to infer that incorporation with alkali carbonate can also make modification of band structure for ceria materials. After calculation, the band gap of SDC–carbonate system shows a visible red shift compared to that of pure ceria (3.2 eV) [79]. In the next section, we will discuss the possible mechanisms on superionic conduction of the ceria–carbonate systems from the perspective of phase junction.

#### Phase Junction of Ceria–Carbonate CHC Systems

Generally, there are two cases of phase junction for ceria–carbonate CHC systems. Here, pure ceria phase is called phase A and the carbonate-incorporated phase is called phase B. The incorporated carbonate will decrease the energy band structure of phase B, thus leading to the band alignment between phase A and phase B [79].

Two cases should be considered for the ceria–carbonate CHC systems. I) the core–shell structure as shown in Fig. 10. The amorphous carbonate phase will accumulate on the surface of bulk ceria and permeate into the superficial ceria phase, resulting in a core–shell structure. The internal pure ceria phase (phase A) remains the initial energy band structure, while the energy band structure of the superficial carbonate-incorporated ceria phase (phase B) will decrease, which leads to the band alignment and resultant BIF (Fig. 10). Meanwhile, the interface can also generate superionic highways for ionic shuttles.

**Fig. 13:**
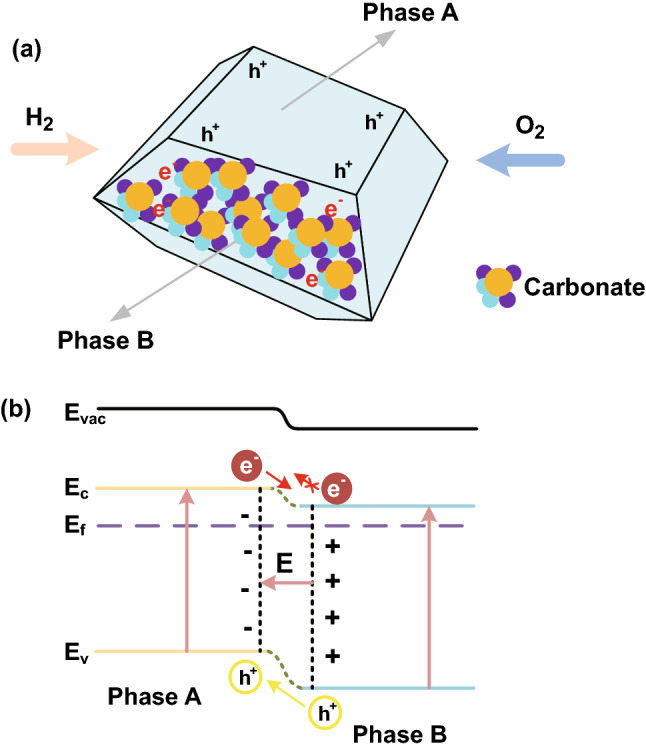
Illustration of **a** formation of phase junction in ceria–carbonate CHC system; **b** band alignment and BIF in ceria–carbonate CHC system

Figure 13a displays another case by forming phase junction in the ceria–carbonate CHC system, i.e., bare ceria particles (phase A) and carbonate-incorporated ceria phase (phase B). As mentioned before, a phase junction can generate between these two phases, resulting in a spatial separation of electron–hole pairs (shown in Fig. 13b).

In conclusion, phase junction is a reasonable mechanism for the enhanced conductivity of ceria–carbonate CHC systems. Similar to the *p*–*n* junction, phase junction helps to facilitate the separation of electron–hole pairs. Simultaneously, the proper band alignment and corresponding BIF contribute to block the migration of electrons as well as promote ionic transport. Therefore, ceria–carbonate CHC possesses remarkable ionic conductivity and performance.

## Conclusions and Prospects

CHC material systems have been an area of interest for the LTSOFC research and development with global impact and activities because of their excellent properties and device functions, e.g., superionic conductivity and excellent fuel cell performances. In this review, we have summarized interfacial effects and the mechanisms of multi-ionic conduction of CHCs. Most importantly, we have introduced band theory and emphasized the importance of band alignment and BIF between constituent phases in the CHC systems to deepen the understanding and present new scientific principles of superionic conduction in CHCs.

The “high transport pathways” can be designed by band theory and are achievable by constructing heterostructures in the CHC systems. The conventional interfacial effects to explain this phenomenon from a mechanical perspective, where the formation of the “high transport pathways” is attributed to the mismatch between two lattices. However, it is not enough to explain the superionic conduction comprehensively. Thus, the concepts of the band alignment and BIF have been introduced.

Compared with the conventional interfacial mechanical explanation, band alignment and BIF may provide another methodology to describe superionic conduction. The effects of the band alignment and BIF on superionic conduction can be understood from below aspects:

i. BIF can decrease the activation energy thus leading to significantly reduced barrier for the migration of ions in the CHC systems.
ii. Increase in the mobile ions concentration due to strong BIF, which can activate ions from static to mobile state, so that more mobile ions can be transported. As a result, the conductivity of ions will increase.
iii. BIF can promote ionic migration at the interface in the CHCs [80].

Therefore, the band bending and BIF have presented a new scientific understanding and principle for superionic conduction in CHCs.

In addition to the above, superionic conduction should fulfill a number of conditions:

1. A large interfaces and surfaces in constituent phases, where nanoscale phase materials are favorable due to providing a high pathway framework for superionic conduction.
2. Proper energy band alignment between the constituent phases, which avoids the internal short circuit by forming BIF.
3. The formation of the BIF can induce and assist the migration of the ions from the above three aspects.

As of now, functional ceria–semiconductor CHCs have been widely used in the fuel cells. One of the most exciting examples can be taken is that a *p*-type Na_*x*_CoO_2_/CeO_2_ (*n*-type) CHC material enables super protonic conduction 0.3 S cm^−1^ at 520 °C, resulting in a fuel cell power output > 1000 mW cm^−2^. These outstanding results are attributed to BIF induced metallic state leading to confined protons interfacial highways [80]. Energy band theory and BIF provide a very different approach to study and deeply understand the internal mechanism, which can be a broader methodology to design material functionalities and understanding of the material properties. Further, it can be predicted that combining the EB theory with the CHC systems would speed up research and development as well as extending applications for new generation fuel cells.

## Appendix

### Born–Oppenheimer Approximation and Hartree–Fock Equation

In quantum mechanics, the Schrödinger equation of the system is required to describe a multi-particle system [81]:

27 $$H\varphi (r,R) = E^{H} \varphi (r,R)$$

28 $$H(r,R) = - \mathop \sum \limits_{i} \frac{{h^{2} }}{{2m}}\nabla _{{r_{i} }}^{2} + \frac{1}{2}\mathop \sum \limits_{{i,i^{\prime}}} '\frac{{e^{2} }}{{\left| {r_{i} - r_{i} '} \right|}} - \mathop \sum \limits_{j} \frac{{h^{2} }}{{2M_{j} }}\nabla _{{R_{i} }}^{2} + \frac{1}{2}\mathop \sum \limits_{{j,j^{\prime}}} '\frac{{Z^{2} e^{2} }}{{\left| {R_{j} - R_{j} '} \right|}} - \mathop \sum \limits_{{i,j}} \frac{{Ze^{2} }}{{\left| {r_{i} - R_{j} } \right|}}$$

where *R*_*j*_ refers to the potential vectors of electrons, *r*_*i*_ refers to the potential vectors of nuclei, *M*_*j*_ refers to the mass of nucleus and *m* refers to the mass of electrons. It can be observed from the above formula: the first term represents the kinetic energy of electrons, the second term represents the interaction between electrons, the third term represents the kinetic energy of nucleus, the fourth term represents the Coulomb interaction between atoms and the last term represents the Coulomb interaction between electrons and nucleus.

Considering that the outermost valence electrons in the atom play a major role in the solid physics problem, the rest electrons and the nucleus together form an ion core. The mass of these electrons is classified into* M*_*j*_, and the value of* Z* is appropriately adjusted. Since the mass of the ion core is much larger than that of the electrons, the characteristic velocity is much slower than that of the electrons, so that the Born–Oppenheimer approximation [82] can be used to separate the motion of the ions and electrons. Therefore, the Hamilton quantity* H* of the electronic system under adiabatic approximation can be simplified to the following formula:

29 $$H(r,R) = - \mathop \sum \limits_{i} \frac{{h^{2} }}{{2m}}\nabla _{{r_{i} }}^{2} + \frac{1}{2}\mathop \sum \limits_{{i,i^{\prime}}} '\frac{{e^{2} }}{{\left| {r_{i} - r_{i} '} \right|}} - \mathop \sum \limits_{{i,j}} \frac{{Ze^{2} }}{{\left| {r_{i} - R_{j} } \right|}}$$

However, due to the existence of Coulomb interaction term, it is still difficult to obtain accurate solutions. In fact, the Coulomb interaction term cannot be completely ignored, but an appropriate correction term can be introduced to modify the formula. Thus, Hatree's self-consistent field was introduced to describe the electron interaction potential:

30 $$V_{i} (r_{i} ) = \mathop \sum \limits_{{i^{\prime}(i^{\prime} \ne i)}} \int {\mathrm{d}}r'\frac{{\left| {\varphi _{i} '(r^{\prime})} \right|^{2} }}{{\left| {r^{\prime} - r} \right|}}$$

With the Hatree's self-consistent field, Schrödinger equation can be rewritten into:

31 $$\left\{ {E - \mathop \sum \limits_{{i = 1}}^{N} [H_{0} (r_{i} ) + V_{i} (r_{i} )]} \right\}\varphi _{i} (r_{i} ,r_{2} \ldots r_{N} ) = 0$$

Assuming that the wave function can be expressed as the continuous product of the single electron wave function, the above formula can be decomposed into the equations satisfied by single electron wave functions:

32 $$\left[ {H_{0} \left( r \right) + V_{i} \left( r \right) - E_{i} } \right]\varphi _{i} \left( r \right) = 0$$

If* φ*_*i*_ is settled, Eq. (6) can be solved by the self-adaptive method, and the total energy of the electronic system could be described as:

33 $$E_{e} = \mathop \sum \limits_{i} E_{i} - \frac{1}{2}{\iint }\frac{{\rho \left( r \right)\rho \left( {r^{\prime}} \right)}}{{\left| {r - r^{\prime}} \right|}}{\mathrm{d}}r{\mathrm{d}}r'$$

And the charge density here is:

34 $$\rho (r) = \sum \left| {\varphi _{i} (r)} \right|^{2}$$

If the exchange energy term (caused by electrons of each pair of parallel spins) is added, the total energy represented by the above formula can be rewritten into:

35 $$E_{x} = \frac{1}{2}\mathop \sum \limits_{{i \ne i^{\prime}}} {\iint }\varphi _{i} (r)\varphi _{{i^{\prime}}} (r') \times \frac{1}{{\left| {r - r^{\prime}} \right|}}\varphi _{i} (r')\varphi _{{i^{\prime}}} (r){\mathrm{d}}r{\mathrm{d}}r'$$

In this way, Hartree–Fock equation is obtained [83].

### Electron Density Functional Theory

Thomas [84] and Fermi [85] proposed that electrons in a multi-electron system could be regarded as a uniform free electron gas system, where electronic kinetic energy, nucleus–electron Coulomb interaction and electron–electron Coulomb interaction can be expressed as a function of single variable electron density. However, it failed to take the electronic exchange interaction into account and the accuracy of the model is limited.

Later, Dirac et al*.* [86] added the local density approximation (LDA) of electron exchange interaction and acquired the energy functional expression of electrons in external potential *V*_ext_(r):

36 $$E_{{TF}} (\rho ) = C_{1} \int {\mathrm{d}}^{3} r\rho (\vec{r})^{{{\raise0.7ex\hbox{$5$} \!\mathord{\left/ {\vphantom {5 3}}\right.\kern-\nulldelimiterspace} \!\lower0.7ex\hbox{$3$}}}} + \int {\mathrm{d}}^{3} rV_{{{\mathrm{ext}}}} (\vec{r})\rho (\vec{r}) + C_{2} \int {\mathrm{d}}^{3} r\rho (\vec{r})^{{{\raise0.7ex\hbox{$4$} \!\mathord{\left/ {\vphantom {4 3}}\right.\kern-\nulldelimiterspace} \!\lower0.7ex\hbox{$3$}}}} + \frac{1}{2}\int {\mathrm{d}}^{3} r{\mathrm{d}}^{3} r'\frac{{\rho (\vec{r}) - \rho (\vec{r}')}}{{\vec{r} - \vec{r}'}}$$

where the first term is the LDA expression of kinetic energy, the second term is the external potential interaction energy, the third term is the expression of exchange interaction and the fourth term is Coulomb interaction energy.

Afterward, Hohenberg and Kohn proposed density functional theory (DFT), which is based on electron density, not all-electron wave function [87]. They considered that the electron density could be used as a basic variable to determine all ground-state properties of multi-electron systems in an external potential field. Herein, an imaginary non-interacting electronic system composed of multiple orbitals $\varphi _{i} \left( {\vec{r}} \right)$ with a single electron was constructed, with the condition that the non-interacting and interacting systems have the same electron density $\rho (\vec{r})$:

37 $$\rho (\vec{r}) = \mathop \sum \limits_{i} f_{i} \sum \left| {\varphi _{i} (\vec{r})} \right|^{2}$$

where *f*_*i*_ is the occupation number of orbitals. The energy of an interacting electronic system was written as a function of electron density:

38 $$E\left[ \rho \right] = T_{0} \left[ \rho \right] + E_{{{\mathrm{ext}}}} \left[ \rho \right] + E_{{\mathrm{H}}} \left[ \rho \right] + E_{{{\mathrm{XC}}}} \left[ \rho \right]$$

where kinetic energy is:

39 $$T_{0} \left[ \rho \right] = \mathop \sum \limits_{i} f_{i} \int \varphi _{i}^{*} (\vec{r})\left[ {\frac{{ - h^{2} }}{{2m}}\nabla ^{2} \varphi _{i} (\vec{r})} \right]{\mathrm{d}}\vec{r}$$

Energy related to external potential energy is:

40 $$E_{{{\mathrm{ext}}}} = \int {\rho (\vec{r})V_{{{\mathrm{ext}}}} (\vec{r}){\mathrm{d}}\vec{r}}$$

where *E*_H_ is related to Hartree potential energy and *E*_XC_ refers to exchange–correlation energy. If $\varphi _{i} (\vec{r})$ can be modified, these orbitals should satisfy the Schrödinger equation of a single electron to minimize the energy *E* of the system:

41 $$\frac{{ - h^{2} }}{{2m}}\nabla ^{2} \varphi _{i} (\vec{r}) + V_{{{\mathrm{ext}}}} (\vec{r})\varphi _{i} (\vec{r}) + V_{{\mathrm{H}}} \varphi _{i} (\vec{r}) + V_{{{\mathrm{XC}}}} \varphi _{i} (\vec{r}) = \varepsilon _{i} \varphi _{i} (\vec{r})$$

where the exchange–correlation potential *V*_XC_ is:

42 $$V_{{{\mathrm{XC}}}} (\vec{r}) = \frac{{\delta E_{{{\mathrm{XC}}}} }}{{\delta \rho (\vec{r})}}$$

Considering that the specific calculating processes based on the above fundamental equations of DFT are much complex, a lot of codes or programs have been developed. And here, first, the periodic CRYSTAL14 code was selected to perform the DFT calculation [88]. Then, the effective core potential (ECP) was introduced, where different elements could be chosen with different ECPs. Also, basis sets were used for the valence electrons of elements and other atoms.
